# Gestational trophoblastic neoplasia with pancreatic metastasis: clinical characteristics, treatment strategies, and outcomes

**DOI:** 10.1186/s13023-025-04014-6

**Published:** 2025-09-24

**Authors:** Xinghan Cheng, Dan Wang, Xiaoyu Wang, Yang Gui, Xiaoyan Chang, Fengzhi Feng, Jun Zhao, Junjun Yang, Yang Xiang

**Affiliations:** 1 Department of Obstetrics and Gynecology, National Clinical Research Center for Obstetric & Gynecologic Diseases, Peking Union Medical College Hospital, Chinese Academy of Medical Sciences & Peking Union Medical College, 1 Shuaifuyuan, Wangfujing, Dongcheng District, Beijing, 100730 China; 2 Department of Ultrasound, Peking Union Medical College Hospital, Chinese Academy of Medical Sciences & Peking Union Medical College, Beijing, China; 3 Department of Pathology, Peking Union Medical College Hospital, Chinese Academy of Medical Sciences & Peking Union Medical College, Beijing, China

**Keywords:** Gestational trophoblastic neoplasia, Choriocarcinoma, Placental site trophoblastic tumor, Epithelioid trophoblastic tumor, Pancreas, Metastasis

## Abstract

**Objective:**

Pancreatic metastasis of gestational trophoblastic neoplasia (GTN) is extremely rare, with only a few reported cases.

**Methods:**

A retrospective analysis was conducted on GTN patients with pancreatic metastasis at Peking Union Medical College Hospital (2000–2024) and a literature review was performed. A descriptive analysis was carried out on the clinical characteristics, treatment strategies, and outcomes of patients who met the inclusion criteria. Fisher's exact test was used to analyze differences in metastatic patterns and clinical outcomes among patients with different clinical characteristics.

**Results:**

A total of 24 cases were identified (7 from our institute, 17 from literature): 18 choriocarcinomas, 5 placental-site trophoblastic tumors, and 1 epithelioid trophoblastic tumor. Pancreatic metastasis led to organ-specific symptoms. Treatments included chemotherapy (single/multi-agent), immunotherapy, and targeted therapy. Six patients underwent surgical or localized interventions. Outcomes varied: 9 (37.5%) achieved disease-free survival, 5 (20.8%) had partial remission, and 10 (41.7%) died. Surgical or invasive interventions were associated with significantly improved outcomes (*P* = 0.024).

**Conclusion:**

Pancreatic invasion in GTN is a high-risk condition often associated with poor outcomes. Advanced imaging techniques enhance diagnostic accuracy, while endoscopic ultrasound-guided fine-needle biopsy provides essential histopathological confirmation. Multi-agent chemotherapy remains the cornerstone of treatment, with surgical interventions carefully tailored to the individual patient’s condition. For better management and prognosis, an initial treatment strategy integrating multi-agent chemotherapy, immunotherapy, and targeted therapies may offer benefits; however, further investigation is warranted.

## Introduction

Gestational trophoblastic disease comprises a heterogeneous group of premalignant to malignant conditions that arise from the villous trophoblasts of the placenta. The malignant forms of gestational trophoblastic disease are collectively termed gestational trophoblastic neoplasia (GTN), which include invasive moles, choriocarcinoma, placental site trophoblastic tumor (PSTT), and epithelioid trophoblastic tumor (ETT). Among the rare variants of GTN, intraplacental choriocarcinoma comprises approximately 2% of reported cases [1–3]. The treatment of GTN primarily involves chemotherapy and surgery. Choriocarcinoma exhibits a high sensitivity to chemotherapy, with cure rates surpassing 94%, even in patients with advanced-stage disease [4].

Due to the affinity of the trophoblast cells for blood vessels, distant metastasis can occur through hematogenous dissemination even in the early stages. Statistics show that about 50–60% of choriocarcinoma patients present with distant metastases at their initial diagnosis. The most common metastatic sites are the lungs (80%), vagina (30%), pelvis (20%), liver (10%), and brain (10%) [5]。Pancreatic metastasis of GTN is an extremely rare clinical scenario, typically associated with poor prognosis, and only a few cases have been reported in the literature. Due to its rarity, the prognosis and clinical characteristics of pancreatic metastasis remain to be clarified. In this study, we summarize the clinical characteristics, treatment strategies, and outcomes of GTN patients with pancreatic invasion to enhance understanding of the disease and guide clinical management. Additionally, we conducted a comprehensive review of the existing literature related to this condition.

## Materials and methods

### Study design

A retrospective study of GTN patients diagnosed with pancreatic metastasis in Peking Union Medical College Hospital was performed. Eligible patients were diagnosed with pancreatic metastases via imaging or histopathology, regardless of the stage of disease, during the study period (between January 2000 and October 2024) and were aged ≥ 18 years at the time of diagnosis. Clinical information was retrieved and systematically organized from the hospital's electronic medical record system. Additional information was obtained through telephone contact when necessary. Patients were staged according to the revised International Federation of Gynecology and Obstetrics (FIGO) criteria for GTN, and FIGO prognostic scores were assigned where applicable. Complete remission was defined as three consecutive weekly measurements of normal beta-serum human chorionic gonadotropin (β-hCG) levels. Partial remission was diagnosed when β-hCG levels declined logarithmically or the tumor focus decreased in size by more than 50%. Disease progression was defined as a sustained plateau or increase in β-hCG levels or the emergence of new metastatic lesions.

A comprehensive search of the PubMed, Web of Science, and Embase databases was conducted using MeSH terms in MEDLINE and Emtree terms in Embase. The following keywords were used: gestational trophoblastic neoplasia, choriocarcinoma, placental-site trophoblastic tumor, epithelioid trophoblastic tumor, pancreas, metastasis. Studies reporting pancreatic metastasis from GTN with supporting histological and/or imaging evidence were included. Reports with incomplete clinical data, as well as conference abstracts and posters, were excluded. Two authors independently screened all titles and abstracts, followed by a review of relevant articles. Discrepancies were resolved through consensus or consultation with a third reviewer. The quality of the included studies was assessed using the Joanna Briggs Institute (JBI) Critical Appraisal Checklist for Case Reports [6].

### Statistical analyses

Fisher’s exact test was used to analyze differences in metastatic patterns and clinical outcomes across patient subgroups, due to small sample sizes and expected cell counts of less than 5. Statistical analyses were conducted using SPSS software (version 22.0; IBM Corp., Armonk, NY). All tests were two-sided, and a P-value < 0.05 was considered statistically significant.

### Ethics statement

This study was approved by the Institutional Review Board of Peking Union Medical College Hospital (I-23PJ461). Written informed consent was obtained from all participants.

## Results

### Case series

**Fig. 1:**
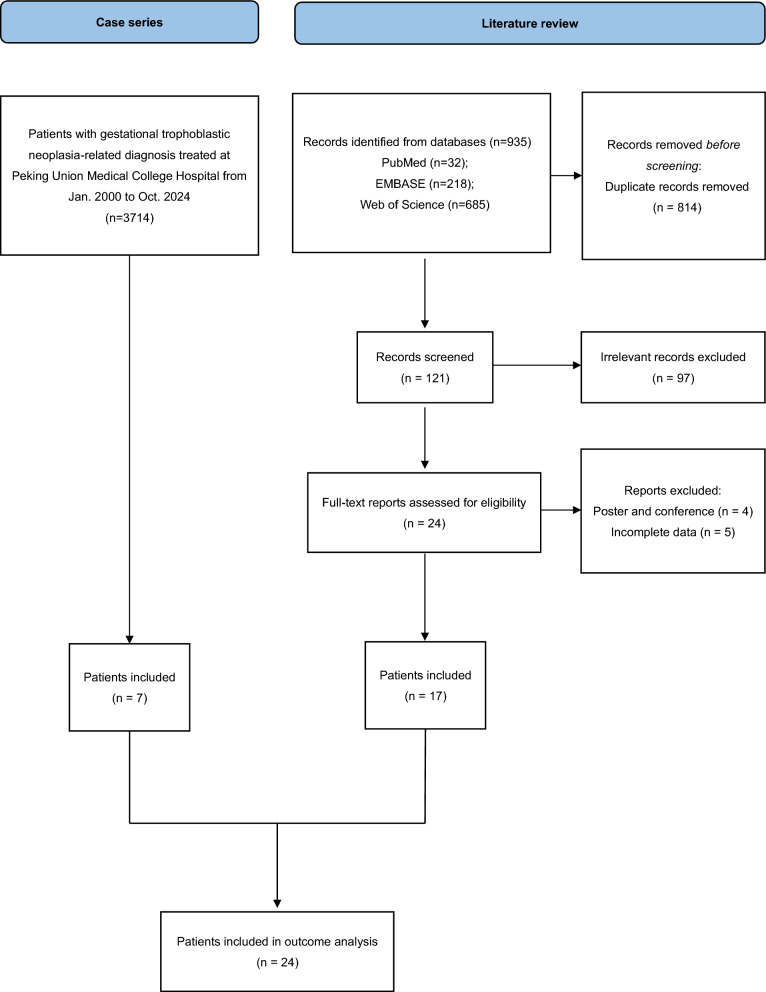
Flow diagram for the selection of included patients

Seven patients from our institute were included in the study. Clinical characteristics, treatment strategies, and outcomes of the patients are summarized in Table 1. During this period, 3714 patients with GTN related diagnosis were retrieved from electronic medical record system. For the literature review, 935 potentially relevant studies were identified. After removing duplicates, 121 studies remained for title and abstract screening, with 97 irrelevant records subsequently excluded. Full-text reviews were then conducted for the remaining 24 articles. For each study, data on the first author, year of publication, country of origin, tumor type, metastatic sites, clinical symptoms, pre-treatment β-hCG levels, treatment modalities, and outcomes were extracted and are presented in Table 2. The quality assessment checklist of included studies is shown in Table 3**.** The study selection process is illustrated in the flowchart shown in Fig. 1.

**Table 1:** Summary of the characteristics of cases in our institute

Patient	Age	Tumor type	Metastatic site	Pretreatment β-hCG (IU/L)	Antecedent pregnancy	Interval (Months)	FIGO stage	Prognostic score	Initial symptoms	Treatment	Outcome
A	34	CC	Pancreas, lungs, liver, brain	12,185	Abortion	10	IV	14	Hemoptysis	Thoracoscopic left lower lobectomy, AE*2 cycles, EMA/CO + Camrelizumab*6 cycles, oral Apatinib, Camrelizumab*4 cycles, FAEV*4 cycles, EMA/CO + Toripalimab*2 cycles, TP/TE + Toripalimab*1 cycle, hepatic artery infusion of Fluorouracil + AEV + Bevacizumab*3 cycles	Died of gastrointestinal bleeding
B	53	PSTT	Pancreas, lungs	619	Term	276	IV	-	Vaginal bleeding	Hysterectomy + bilateral salpingo-oophorectomy, FAEV*3 cycles, TP/TE*5 cycles	Complete remission
C	44	CC	Pancreas, brain, liver, kidney, breast	63,506	Term	156	IV	16	Vaginal bleeding	Right breast lumpectomy, multiple regimens of chemotherapy*49 cycles	Died of cerebral hemorrhage
D	32	PSTT	Pancreas, liver	2280	Term	8	IV	-	Vaginal bleeding	Hysterectomy, multiple regimens of chemotherapy*31 cycles, including hepatic artery infusion* 1 cycle	Died of gastrointestinal bleeding
E	40	CC	Pancreas, cervix, lungs, brain	268,541	Term	2	IV	14	Abdominal pain, syncope	Cervical mass excision during a cesarean section, AE*2 cycles, FAV*1 cycle	Died of respiratory and circulatory failure
F	24	CC	Pancreas, liver, kidney, lungs	14,000	Term	14	IV	20	Vaginal bleeding	AE*1 cycle, EMA-CO*4 cycles, EMA-EP*1 cycle, PEA*1 cycle, Fluorouracil intraperitoneal arterial infusion*1 cycle, FAEV*2 cycles	Disease progression
G	28	CC	Pancreas, liver, lungs, adrenal gland	10,178	Term	12	IV	16	Vaginal bleeding	FMEV*2 cycles, EMA-CO*4 cycles, TP/TE*2 cycles	Disease progression

β-hCG: beta-serum human chorionic gonadotropin; FIGO: International Federation of Gynecology and Obstetrics; CC: choriocarcinoma; PSTT: placental-site trophoblastic tumor; AE: actinomycin D and etoposide; FAEV: fluorouracil, actinomycin D, etoposide, and vincristine; FMEV: fluorouracil, methotrexate, etoposide, and vincristine; EMA-CO: etoposide, methotrexate, actinomycin D, and vincristine; TP/TE: paclitaxel + cisplatin / paclitaxel + etoposide; AEV: actinomycin D, etoposide, and vincristine; PEA: cisplatin, etoposide, and actinomycin D

**Table 2:** A compilation of reported cases of pancreatic invasion from GTN

References	Country	Age	Tumor Type	Tumor Site	Pretreatment β-hCG (IU/L)	Symptom	Treatment	Outcome
Coşkun [7]	Turkey	30	CC	Pancreas, liver	8800	Abdominal tenderness, mild distention, fever, intraperitoneal hemorrhage	Surgical hemostasis	Referred to another hospital for chemotherapy
Alvarez [8]	USA	24	CC	Pancreas, liver, lungs	9073	Vaginal bleeding, lower abdominal pain, nausea, and vomiting	MTX*4 cycles, ACTD*5 cycles, EMA-CO, en bloc distal pancreatectomy/splenectomy, and adrenalectomy	Plan to begin further chemotherapy
Cheng [9]	China	44	ETT	Pancreas, lungs	21,782	Missed periods, irregular vaginal bleeding	Hysterectomy, EMA-CO*5 cycles	Plan to begin further chemotherapy
Ayas [10]	Turkey	24	PSTT	Pancreas, liver, lungs, kidneys, breast, adrenal and thyroid gland	23,624	Intermittent vaginal bleeding	Hysterectomy and pelvic lymph node dissection, EMA-CO*6 cycles, BEP*4 cycles	Complete remission
Ramachandran [11]	India	27	CC	Pancreas, jaw	98,000	Abdominal pain, fullness, swelling jaw	MTX	Regular follow-up
Rao [12]	China	28	CC	Pancreas	1004	Irregular vaginal bleeding	Partial pancreatectomy, 2 cycles of chemotherapy	hCG return to normal five weeks after surgery, doing well 34 months after surgery
Fatema [13]	Oman	30	CC	Pancreas, liver	49,200	Fever, abdominal pain, intraperitoneal hemorrhage, hypovolemic shock	Surgical hemostasis, hepatic artery embolization	Died from hemorrhagic shock
Youn [14]	Korea	47	CC	Pancreas, liver, lungs, brain	225,000	Abdominal pain, nausea, loss of appetite, headache, weakness, left visual field loss	Conservative treatment	Died from cerebral edema and hypernatremia 15 days later
Huang [15]	USA	26	CC	Pancreas, liver, lungs, brain	63,000	Shortness of breath, right-sided abdominal pain	EP*1 cycle, EMA-CO*4 cycles, EMA-EP*3 cycles, Pembrolizumab*3 cycles	Chemo-refractory, hCG dramatically decrease after 2 cycles of Pembrolizumab
Seresht [16]	Iran	35	PSTT	Pancreas, liver	220	Continuous vaginal bleeding	MTX, ATCD, EMA-CO, EMA-EP, distal pancreatectomy and splenectomy	Complete remission
Stockton [17]	UK	39	CC	Pancreas, liver, lungs, breast	48,990	Shortness of breath and pleuritic chest pain	EP	Died of respiratory failure
Muktesh [18]	India	27	CC	Pancreas	1649	Severe epigastric pain, abdominal distension, fever, breathlessness	Conservative treatment	Died from severe acute pancreatitis, pulmonary thromboembolism, and bronchopneumonia
Yin [19]	China	43	PSTT	Pancreas, liver	148	Recurrent syncope, continuous abdominal pain	FAV*5 cycles, FAEV*1 cycle, hepatic artery embolization, EMP*4 cycles, EMA-CO*2 cycles, partial pancreatectomy, TP*5 cycles	Follow up to 18 months without any signs of recurrence
Sunagozaka [20]	Japan	43	CC	Pancreas, liver, lungs, adrenal gland, lumber vertebra	220	Irregular vaginal bleeding	EMA-CO*11 cycles, preventive pancreatic aneurysms embolization	Complete remission
Zhang [21]	China	46	CC	Pancreas, liver	59,283	Epigastric discomfort	^125^I-seed implantation, transcatheter arterial chemoembolization, and EMA-CO *3 cycles	β-hCG decrease to 348.3 three months after diagnosis
Kumar Upadhyay [23]	India	24	CC	Pancreas, brain, kidneys, lungs, liver, small bowel	1165	Headaches, seizures, and weakness	Whole-brain radiotherapy, EMA-CO*7 cycles, EMA-EP*1 cycle, TP/TE*4 cycles, GEMCAP*5 cycles	β-hCG decrease to 73.2
Dai [22]	China	30	CC	Pancreas, liver, lungs, kidney	480	Abdominal pain, hemoptysis	Chemotherapy	Responded well to chemotherapy, under regular follow-up

β-hCG: beta-serum human chorionic gonadotropin; CC: choriocarcinoma; PSTT: placental-site trophoblastic tumor; ETT: epithelioid trophoblastic tumor; MTX: methotrexate; ACTD: actinomycin D; FAEV: fluorouracil, actinomycin D, etoposide, and vincristine; EMA-CO: etoposide, methotrexate, actinomycin D, and vincristine; EP: etoposide and cisplatin; EMA-EP: etoposide, methotrexate, actinomycin D, and cisplatin; FAV: floxuridine, actinomycin D, vincristine; EMP: etoposide, methotrexate, platinum; TP: paclitaxel and cisplatin; BEP: bleomycin, etoposide, cisplatin; GEMCAP: gemcitabine, capecitabine

**Table 3:** JBI Critical Appraisal Checklist for Reviews

References	Study type	D1	D2	D3	D4	D5	D6	D7	D8
Coşkun [7]	Case report	★	★	★	★				★
Alvarez [8]	Case report	★	★	★	★	★	★		★
Cheng [9]	Case series	★	★	★	★	★	★	★	★
Ayas [10]	Case report	★	★	★	★	★	★	★	★
Ramachandran [11]	Case report	★	★	★	★	★			★
Rao [12]	Case report	★	★	★	★	★	★		★
Fatema [13]	Case report	★	★	★	★	★	★	★	★
Youn [14]	Case report	★	★	★	★	★	★	★	★
Huang [15]	Case report	★	★	★	★	★	★	★	★
Seresht [16]	Case report	★	★	★	★	★	★		★
Stockton [17]	Case report	★	★	★	★	★	★	★	★
Muktesh [18]	Case report	★	★	★	★	★	★	★	★
Yin [19]	Case report	★	★	★	★	★	★	★	★
Sunagozaka [20]	Case report	★	★	★	★	★	★	★	★
Zhang [21]	Case report	★	★	★	★	★			★
Kumar Upadhyay [23]	Case report	★	★	★	★	★	★	★	★
Dai [22]	Case report	★	★	★	★	★			★

JBI Critical Appraisal Checklist for Case Reports. D1. Were patients’ demographic characteristics clearly described? D2. Was the patient’s history clearly described and presented as a timeline? D3. Was the current clinical condition of the patient on presentation clearly described? D4. Were diagnostic tests or assessment methods and the results clearly described? D5. Was the intervention(s) or treatment procedure(s) clearly described? D6. Was the post-intervention clinical condition clearly described? D7. Were adverse events (harms) or unanticipated events identified and described? D8. Does the case report provide takeaway lessons?

**Fig. 2:**
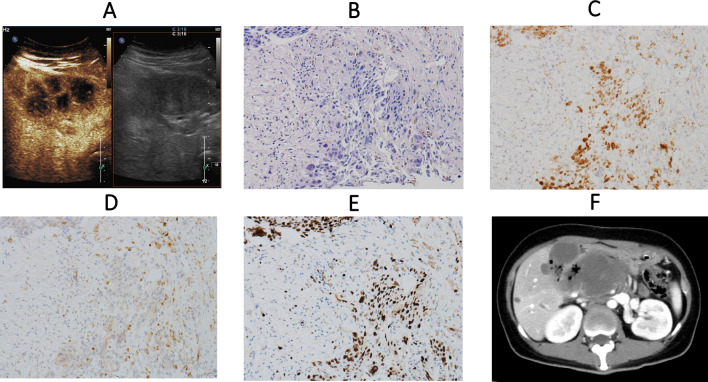
The imaging and histopathological presentations of Patient A. **A** Contrast-enhanced ultrasound showed a hypoechoic lesion measuring 4.8 × 7.3 × 4.4 cm in the pancreatic head. The lesion appears lobulated with clear boundaries and contains abundant strip-like blood flow signals. **B** Pathology showed clusters of atypical cells. **C** Immunohistochemistry staining of SALL-4. **D** Immunohistochemistry staining of HCG. **E** Immunohistochemistry staining of Ki-67. **F** Enhanced abdominal CT showed that the pancreatic head mass significantly enlarged, with blurred boundaries, and the adjacent duodenal lumen is invaded

The median age of the enrolled patients from our institute was 36.4 years (range: 24–53 years). Among them, five were diagnosed with choriocarcinoma, with an average prognostic score of 16, classifying them as ultra-high-risk cases. The remaining two patients were diagnosed with PSTT. In addition to pancreatic involvement, common co-existing metastatic sites included the liver (5/7), lungs (5/7), brain (3/7), and kidneys (2/7). Pretreatment serum β-hCG levels ranged from 619 to 268,541 IU/L, with five patients presenting levels exceeding 10,000 IU/L. Regarding previous obstetric history, six patients had a prior term delivery, while one had a history of abortion. Three patients presented with pancreas-related symptoms, such as abdominal pain, jaundice, nausea, and melena, and two patients experienced severe hematemesis. Diagnosis of pancreatic lesions in these seven cases was achieved through ultrasound, abdominal computed tomography (CT), and/or positron emission tomography/computed tomography (PET/CT). One patient had histopathological confirmation of pancreatic metastases via endoscopic ultrasound-guided fine-needle biopsy (EUS-FNB). All patients underwent multi-agent chemotherapy. For patients diagnosed with choriocarcinoma, induction chemotherapy with the AE regimen (actinomycin D and etoposide) was administered, followed by multi-agent chemotherapy regimens including EMA/CO (etoposide, methotrexate, actinomycin D, cyclophosphamide, and vincristine), FAEV (fluorouracil, actinomycin D, etoposide, and vincristine), or FAV (floxuridine, actinomycin D, and vincristine). For patients diagnosed with PSTT, treatment included regimens such as FAEV and BEP (bleomycin, etoposide, and cisplatin). For those with resistant or recurrent disease, salvage therapies included EMA-EP (etoposide, methotrexate, actinomycin D, and cisplatin), TP/TE (paclitaxel plus cisplatin or paclitaxel plus etoposide), and VIP (etoposide, ifosfamide, and cisplatin). Due to a high prognostic score, one patient (Patient A) was enrolled in a clinical trial and received a combination therapy comprising multi-agent chemotherapy, immunotherapy, and targeted therapy. Two patients underwent arterial infusion chemotherapy. None of the patients underwent surgical interventions for the pancreas following evaluation by the surgical team. Among the seven cases, two patients died from upper gastrointestinal bleeding, one from cerebral hemorrhage, and one from respiratory and circulatory failure. Another two patients discontinued treatment due to disease progression. Only one patient achieved complete remission. Notably, Patient A, presented with metastasis in lungs and brains and a high prognostic score of 14, initially received two cycles of AE introduction chemotherapy, followed by a combination regimen of EMA-CO, Camrelizumab, and oral Apatinib. Her β-hCG levels normalized after six cycles of treatment. However, two months after completing chemotherapy, the disease recurred, primarily manifesting as the newly developed liver and pancreatic mass accompanied by symptoms including abdominal pain, nausea, and vomiting. Magnetic resonance imaging (MRI) findings indicated a mass in the pancreatic head measuring approximately 30 mm, raising the suspicion of a solid pseudopapillary neoplasm. Contrast-enhanced ultrasound was utilized for the differential diagnosis (Fig. 2A), and EUS-FNB of the pancreatic head mass confirmed a diagnosis of metastatic choriocarcinoma (Fig. 2B-E). She was restarted on multi-drug chemotherapy with regimens including FAEV, EMA-CO, and TP/TE, combined with Toripalimab and intrathecal methotrexate. Unfortunately, her response to therapy was inadequate, with disease progression characterized by rising β-hCG levels, recurrent episodes of pancreatitis, and progressive enlargement of the pancreatic mass. In the end, abdominal CT scans indicated the pancreatic head mass invaded the lumen of the duodenum (Fig. 2F), and uncontrolled upper gastrointestinal bleeding ultimately resulted in her death. Patient B, in contrast, demonstrated a favorable response to multi-drug chemotherapy and obtained complete remission. She remains in continuous follow-up 51 months after completing treatment. Patients C, D, E, F, and G exhibited similar patterns of disease progression. For these five patients, the pancreatic lesions were unresectable and showed poor responsiveness to multi-drug chemotherapy.

### Literature review

Seventeen case reports were identified and included from the literature, spanning from 1998 to 2024, involving 17 GTN patients presented with pancreatic invasions [7–23]. The patients ranged in age from 24 to 47, with a median age of 30; most (70.6%) were in their 20 s or 30 s, with a mean age of 33.4. Among the 17 patients, three had pathologically confirmed PSTT, one had ETT, while the remaining eleven had choriocarcinoma. Besides the pancreas, common coexisting metastatic sites included the liver (76.4%), lungs (52.9%), brain (17.6%), kidney (17.6%), breast (11.8%), and adrenal gland (11.8%). Pretreatment serum β-hCG levels varied widely, from 148 to 225,000 IU/L, with 47% showing levels above 10,000 IU/L. Symptoms were generally atypical and varied depending on metastatic sites, including vaginal bleeding, abdominal pain, and nausea. Patients received a range of chemotherapy regimens, including intravenous single- and multi-agent regimens, and transcatheter arterial chemoembolization. In 35.3% of the cases, local invasive treatments targeting the pancreas were employed, such as pancreatectomy, pancreatic aneurysm embolization, and ^125^I-seed implantation. One patient with chemoresistant disease received immunotherapy (Pembrolizumab), resulting in a marked decrease in β-hCG levels and significant clinical improvement. Despite these interventions, the overall survival rate remained low, with 23.5% of patients succumbing to the disease. Of the 17 patients, eight were alive and disease-free, five had partial remission, and the remaining four had died.

A total of 24 patients were included in the metastatic pattern and clinical outcome analysis, comprising seven patients from our study and 17 from previously published literature. Table 4 presents a comparison of metastatic spread patterns based on the histological subtype of the primary tumor. No significant differences were observed in the patterns of metastasis to other organs between choriocarcinoma and PSTT. Table 5 highlights the differences in outcomes based on various clinical characteristics. Notably, a significant difference was observed in patients who received interventions targeting the pancreas (*P* = 0.024), as all six patients undergoing such treatments achieved either complete or partial remission. However, no significant differences were identified in clinical outcomes based on age at diagnosis, tumor type, pretreatment β-hCG levels, the number of metastatic sites, or the use of combined immunotherapy and chemotherapy.

**Table 4:** Metastasis pattern in GTN patients with pancreatic metastasis

Metastatic site	CC	PSTT/ETT	*P*-value
Liver	77.8% (14/18)	66.7% (4/6)	0.618
Lungs	61.1% (11/18)	50% (3/6)	0.665
Brain	33.3% (6/18)	0	0.277
Kidney	22.2% (4/18)	16.7% (1/6)	> 0.999
Breast	11.1% (2/18)	16.7% (1/6)	> 0.999
Adrenal gland	11.1% (2/18)	16.7% (1/6)	> 0.999
Cervix	5.6% (1/18)	0	> 0.999
Jaw	5.6% (1/18)	0	> 0.999
Lumber vertebra	5.6% (1/18)	0	> 0.999
Small bowel	5.6% (1/18)	0	> 0.999
Thyroid gland	0	16.7% (1/6)	> 0.999

CC: choriocarcinoma; PSTT: placental-site trophoblastic tumor; ETT: epithelioid trophoblastic tumor

**Table 5:** Outcome in GTN patients with pancreatic metastasis

Variable	Clinical outcome		*P*-value
	CR or PR	PD	
Age at diagnosis (years)			
< 40	9	7	> 0.999
≥ 40	5	3	
Tumor type			
CC	9	9	0.341
PSTT or ETT	5	1	
Pre-treatment β-hCG (IU/L)			
< 100,000	14	8	0.163
≥ 100,000	0	2	
Number of metastatic sites			
< 3	8	3	0.24
≥ 3	6	7	
Invasive interventions towards pancreas			
Yes	6	0	0.024*
No	8	10	
Treatment strategy			
Chemotherapy	12	6	> 0.999
Chemotherapy + immunotherapy	1	1	

^*^Statistically significant

β-hCG: beta-serum human chorionic gonadotropin; CC: choriocarcinoma; PSTT: placental-site trophoblastic tumor; ETT: epithelioid trophoblastic tumor; CR: complete remission; PR: partial remission; PD: progressive disease

## Discussion

Pancreatic invasion from GTN is an infrequent occurrence, with no available data currently reporting its incidence. Based on statistics from patients treated at our hospital between 2000 and 2024, we estimate that the incidence of pancreatic metastasis in these patients is approximately 0.1–0.2%. However, as our hospital is a tertiary center receiving referrals from across the nation, selection bias must be taken into account. This figure may overestimate the true incidence of pancreatic metastasis in GTN and may not fully reflect real-world conditions. The limitations of imaging examinations may impact the diagnosis of pancreatic metastases, and the application of novel imaging technologies, such as PET/CT, could potentially improve the diagnostic rate. GTN patients with pancreatic involvement are often diagnosed with high-risk, stage IV disease, complicated with multi-organ metastases. Notably, more than half of the enrolled patients had three or more metastatic sites, and the average FIGO prognostic score for patients treated at our institution reached as high as 16. Etoposide-based multi-drug regimens, such as FAEV, EMA-CO, and EMA-EP, remain the cornerstone of treatment for high-risk GTN patients, and induction chemotherapy could provide benefits for patients with poor general conditions [24, 25].

Secondary metastasis to the pancreas is relatively uncommon and often asymptomatic, though it can occasionally present with symptoms such as pain, weight loss, jaundice, acute pancreatitis, or upper gastrointestinal bleeding, which may mimic primary pancreatic cancer. Due to the atypical presentation on imaging, pancreatic metastases can be difficult to distinguish from other benign lesions or primary pancreatic cancer. Ultrasound-guided fine needle aspiration or biopsy is invaluable for obtaining a reliable diagnosis, with accuracy rates reaching up to 97.9% [26]. The most common primary sites for pancreatic metastasis include the kidney, breast, colon, skin, and lung [27]. Pancreatic metastasectomy or pancreatectomy could offer benefits to GTN patients by removing chemotherapy-resistant foci and preventing fatal complications related to the pancreas. Unfortunately, none of the deceased patients in our study underwent pancreatic metastasectomy due to their poor overall condition. Of the 17 cases reviewed, four patients with pancreatic invasion underwent pancreatectomy, and all achieved complete or partial remission [8, 12, 16, 19]. Other invasive treatments, such as ^125^I-seed implantation and pancreatic aneurysm embolization, have also been employed with promising therapeutic outcomes [20, 28]. Although all six patients who underwent invasive interventions targeting the pancreas achieved complete or partial remission, this result must be interpreted with caution, as only patients with good performance status are typically deemed suitable for surgery. Among the six patients who benefited from local invasive treatment, two were diagnosed with PSTT, while the remaining four had choriocarcinoma. Given that choriocarcinoma is generally highly responsive to chemotherapy, whereas PSTT/ETT tends to exhibit a lower response rate, the effectiveness of invasive treatments, including surgical intervention, may vary significantly depending on the pathological type in GTN patients with pancreatic metastases. This discrepancy could also have a considerable impact on clinical decision-making. However, due to the rarity of pancreatic metastases, the currently limited evidence makes it difficult to further clarify this issue.

Immunotherapy, particularly immune checkpoint inhibitors (ICIs) such as anti-programmed cell death protein 1 (PD-1) and its ligand (PD-L1) drugs, has emerged as a novel and effective treatment strategy for GTN. Characterized by a high concentration of paternally derived placental antigens and immune surveillance at the fetal-maternal interface, GTN has demonstrated remarkable responsiveness to immunotherapy. In 2017, Ghorani et al. [29] reported the first successful case of chemotherapy-resistant GTN treated with ICIs, with 75% (3/4) of patients achieving complete remission. A systematic review by Baas et al. encompassing 133 patients, further confirmed the efficacy and low toxicity of ICIs, showing that 65.2% (77/118) of patients with high-risk GTN achieved remission [30]. Additionally, a meta-analysis by Braga et al. demonstrated that Pembrolizumab is effective for treating multidrug-resistant GTN, regardless of histological subtype, with positive responses in cases of choriocarcinoma, PSTT, and ETT [31]. However, the Phase 2 trial TROPHIMMUN (NCT03135769) revealed that the anti-PD-L1 drug Avelumab demonstrated limited efficacy in patients with multidrug-resistant GTN [32]. A multicenter retrospective analysis by Wang et al. revealed that combining ICIs with chemotherapy significantly improved complete remission rates in high-risk or recurrent GTN patients, increasing from 54.3% with monotherapy to 87.1% with combination therapy. Furthermore, salvage chemotherapy proved effective for patients who did not initially respond to ICIs [33]. Retreatment with immunotherapy has also been reported to yield favorable outcomes, with one patient achieving complete remission 24 months post-treatment [34]. Moreover, dual blockade of PD-1 and cytotoxic T lymphocyte-associated antigen 4 (CTLA-4) has demonstrated efficacy in chemotherapy-refractory GTN patients (DART SWOG 1609, NCT03135769) [35]. Among the reviewed cases, Huang et al. [15] described a chemotherapy-resistant GTN patient with multiple metastases (pancreatic head, liver, and lungs) who achieved complete serological remission after two cycles of Pembrolizumab. Unfortunately, in our case (Patient A), ICIs were unsuccessful. Presenting a high prognostic score, Patient A was treated with a combination of multi-agent chemotherapy, immunotherapy, and an antiangiogenic drug. However, relapse occurred only two months post-treatment, and salvage therapy with the combination of immunotherapy and multi-drug chemotherapy was ineffective. The mechanisms underlying acquired resistance to immunotherapy in GTN remain unclear. Possible factors include defects in antigen presentation, loss of neoantigens, and disruptions in interferon signaling [36]. Alternative combination strategies may benefit patients who develop resistance, such as dual immune checkpoint inhibition targeting different pathways, or combining ICIs with multi-drug chemotherapy or targeted therapies [37]. Given the limited treatment options available, further research is crucial to develop effective strategies for GTN patients with immunotherapy resistance.

The main strength of our study lies in being the first and most comprehensive retrospective study to date on pancreatic metastases of GTN, as well as the first to review previously reported cases. However, our study has certain limitations that necessitate cautious interpretation of the findings. This study is based on a retrospective design with a limited sample size, varying treatment modalities, and inconsistent follow-up durations, resulting in significant differences in patient outcomes. Since the aforementioned confounding factors cannot be excluded, making it difficult to draw definitive conclusions from the results. Although randomized controlled trials may represent the ideal study design for evaluating the prognosis of pancreatic metastases in GTN patients, such trials are likely infeasible due to the rarity of this condition.

The clinical significance of this study lies in the detailed presentation of pancreatic metastasis from GTN—an exceptionally rare metastatic pattern—highlighting its clinical manifestations, treatment process, and a preliminary exploration of potential prognostic factors. Surgical resection or localized invasive treatments, when carefully tailored to the appropriate patient population and optimal timing, may be associated with improved clinical outcomes; Emerging combination strategies—including multi-agent chemotherapy, immunotherapy, and targeted therapies—may hold promise for improving outcomes in high-risk patients. However, current evidence is insufficient to draw definitive conclusions. Given the rarity, complexity, and heterogeneity of such cases, referral to specialized trophoblastic disease centers and the involvement of a multidisciplinary team are strongly recommended. Future research should focus on collecting multicenter data and conducting prospective studies to better define optimal management strategies and improve patient prognosis.

In conclusion, pancreatic invasion in GTN is a high-risk condition often associated with poor outcomes. Advanced imaging techniques enhance diagnostic accuracy, while EUS-FNB provides essential histopathological confirmation. Multi-agent chemotherapy remains the cornerstone of treatment, with surgical interventions carefully tailored to the individual patient’s condition. For better management and prognosis, an initial treatment strategy integrating multi-agent chemotherapy, immunotherapy, and targeted therapies may offer benefits; however, further investigation is warranted.

## Data Availability

All data generated or analysed during this study are included in this published article.
